# The Symplectic Camel and Poincaré Superrecurrence: Open Problems

**DOI:** 10.3390/e20070499

**Published:** 2018-06-28

**Authors:** Maurice A. de Gosson

**Affiliations:** Faculty of Mathematics, NuHAG, University of Vienna, 1090 Vienna, Austria; maurice.de.gosson@univie.ac.at

**Keywords:** Poincaré recurrence, symplectic camel, quantum mechanics, Hamiltonian

## Abstract

Poincaré’s Recurrence Theorem implies that any isolated Hamiltonian system evolving in a bounded Universe returns infinitely many times arbitrarily close to its initial phase space configuration. We discuss this and related recurrence properties from the point of view of recent advances in symplectic topology which have not yet reached the Physics community. These properties are closely related to Emergent Quantum Mechanics since they belong to a twilight zone between classical (Hamiltonian) mechanics and its quantization.

## 1. Introduction

In his famous prize-winning 1890 paper [1], Henri Poincaré proved that almost every phase space trajectory of an isolated three-body system must return arbitrarily close to its initial position, and this infinitely many times. Poincaré called this result *théorème de stabilité à la Poisson*, but it is nowadays universally known as *Poincaré’s recurrence theorem*. Poincaré’s theorem lies at the foundations of ergodic theory, and is actually true of any dynamical system moving in a bounded set under the action of measure-preserving transformations. One of the most well known applications of Poincaré’s recurrence is that a bounded Hamiltonian system (“Universe”) must return after some (usually extraordinarily large) time arbitrarily close to its initial configuration. Let us focus on a subsystem of that Universe (a galaxy, or more modestly, the solar system, are good examples). This subsystem will return to its initial configuration after some time—but, what time? If this subsystem does not interact with the rest of the Universe, it will have its own private return time, and it is reasonable to expect (and possible to prove) that this return time is usually shorter than the return time of the whole Universe. Things being what they are, subsystems do usually interact with the rest of the Universe, and it is then no longer reasonable to expect (or possible to prove) that the subsystem will return to its initial configuration before the whole Universe does. The aim of this paper is to briefly and tentatively discuss the possibility of such “superrecurrence” in the presence of interactions: an interacting subsystem of a Hamiltonian system will have its own return time, which is of the same order of magnitude as if there were no interaction. The difficulty lies in the fact that an interacting subsystem of a Hamiltonian system is not Hamiltonian in its own right, but has a much more complicated structure due to the interactions. We use a tool from symplectic topology, related to Gromov’s symplectic non-squeezing theorem (also known as the “principle of the symplectic camel”) which we have abundantly explained and discussed elsewhere [2,3,4,5] (cf. [6]). This theorem plays an essential role in quantum mechanics, and also in the study of entropy [7], aeronautics [8,9], and statistical mechanics [10]. Its importance and potential applications have however not yet been fully exploited in physics. This might be due to the mathematical difficulty of the result, which was only discovered in the mid-1980s by the mathematician Mikhail Gromov, who was awarded the Abel Prize (the equivalent of the Nobel Prize in mathematics) for his discovery. In fact, the principle of the symplectic camel can be seen as an imprint of the uncertainty principle of quantum mechanics in classical mechanics—or the other way around! Without becoming embroiled in a sterile polemic, let me just say that if one considers (as one should do!) quantum theory as the “master theory” of which classical mechanics (in its Hamiltonian formulation) is a macroscopic approximation, then one should find traces of the mathematical structure of quantum theory in the macroscopic domain. This is exactly what happens here: as we have shown in the papers cited above, uncertainty relations (in their strong Robertson–Schrödinger form) are not per se quantum mechanical, but also exist in classical mechanics, but this time for an arbitrary value of Planck’s constant *h*, which is now viewed as a free parameter. It is then the more exact theory (quantum mechanics) which forces us to choose a lower limit for the indeterminacy by fixing a lower bound for the admissible value of the parameter *h* (in more physical terms, it is the quantum phenomena which force us to do so: quantum theory exists only to describe quantum phenomena, and not the other way round). However, more about this is in the Discussion ending the paper.

## 2. Subsystems of Hamiltonian Systems

### 2.1. Description of the Problem

Consider a mechanical system of *N* point-like particles whose motion is determined by a Hamilton function *H*. If the system is confined to a bounded region of phase space $R_{q,p}^{6N}$, Poincaré’s recurrence theorem tells us that any initial pattern of positions and velocities (specified within a given error) will recur, independently of any permutation in the numbering of the particles of the system. The recurrence time is however generally extremely long, (see [11] for a recent analysis of recurrence time), except of course for periodic (or quasi-periodic) systems. Of course, the boundedness condition is essential: a free particle in an infinite Universe will never return to its initial position. Suppose indeed that the system, represented by a phase point $\left(q,p\right)=\left(q_{1},...,q_{N},p_{1},...,p_{N}\right)$, with $q_{i}=\left(x_{i},y_{i},z_{i}\right)$ and $p_{i}=\left(p_{x_{i}},p_{y_{i}},p_{z_{i}}\right)$, is confined to a “universe” U. We are not asking for an exact return of (q,p) but we content ourselves with the return of some (arbitrarily) small neighbourhood Ω of that point. Then, an upper bound for the first return time of that neighbourhood has a magnitude of order T≈Vol(U)/Vol(Ω). This number is usually very large. Let us now focus on a subsystem, identified with a point $\left(q^{\prime },p^{\prime }\right)=\left(q_{1},...,q_{n},p_{1},...,p_{n}\right)$ with n<N. Assume first that the total Hamiltonian function is of the type

(1) $$H=H^{\prime }\left(q^{\prime },p^{\prime }\right)+H^{\prime \prime }\left(q^{\prime \prime },p^{\prime \prime }\right)$$

where $\left(q^{\prime \prime },p^{\prime \prime }\right)=\left(q_{n+1},...,q_{N},p_{n+1},...,p_{N}\right)$. Due to the absence of interaction between the two subsystems $\left(q^{\prime },p^{\prime }\right)$ and $\left(q^{\prime \prime },p^{\prime \prime }\right)$, their motions are independent; the time-evolution of $\left(q^{\prime },p^{\prime }\right)$ is thus governed solely by its own private Hamiltonian $H^{\prime }$; the equations of motions are

(2) $${\dot{q}}_{j}=\frac{\partial H^{\prime }}{\partial p_{j}}\left(q^{\prime },p^{\prime }\right),\;\;{\dot{p}}_{j}=-\frac{\partial H^{\prime }}{\partial q_{j}}\left(q^{\prime },p^{\prime }\right)\;\;\mathrm{with}\;\;1\leq j\leq n$$

and their solutions only dependent on the initial values $q^{\prime }\left(0\right)$ and $p^{\prime }\left(0\right)$. The corresponding universe $U^{\prime }$ consists of the set of all points $\left(q^{\prime },p^{\prime }\right)$ such that $\left(q^{\prime },q^{\prime \prime },p^{\prime },p^{\prime \prime }\right)$ is in U for some $q^{\prime \prime },p^{\prime \prime }$; it is thus the projection of U on the reduced phase space $R_{q^{\prime },p^{\prime }}^{6n}$ and, accordingly, the corresponding neighbourhood $\Omega^{\prime }$ is the projection of Ω on $R_{q^{\prime },p^{\prime }}^{6n}$. Let us now compare the return time $T^{\prime }\approx \mathrm{Vol}\left(U^{\prime }\right)/\mathrm{Vol}\left(\Omega^{\prime }\right)$ for the system $\left(q^{\prime },p^{\prime }\right)$ with that of (q,p). To fix the ideas, we choose for U a hypercube with sides of length *L* and for Ω a hypercube with sides of length ε≪L. It follows that $T\approx {\left(L/\varepsilon \right)}^{6N}$ and that $T^{\prime }\approx {\left(L/\varepsilon \right)}^{6n}$ so that the ratio $T/T^{\prime }$ between both return times is of order ${\left(L/\varepsilon \right)}^{6(N-n)}$. Consider next the general case, where the subsystems interact; we can no longer separate the variables that $q_{i}$ and $p_{i}$; this is the case if for instance,

(3) $$H\left(q,p\right)=\sum_{j=1}^{N}\frac{|p_{j}{|}^{2}}{2m_{j}}+V\left(q_{1},...,q_{N}\right)$$

although everything will hold for an arbitrary function of the variables $q_{j},p_{j}$. We consider again the subsystem $\left(q^{\prime },p^{\prime }\right)$; its motion will now depend on the global behaviour of the system (q,p), since the solutions of the corresponding Hamilton equations

(4) $${\dot{q}}_{j}=\frac{\partial H}{\partial p_{j}}\left(q,p\right),\;\;{\dot{p}}_{j}=-\frac{\partial H}{\partial q_{j}}\left(q,p\right)\;\;\mathrm{with}\;\;1\leq j\leq n$$

now depend on the initial values of *all* variables $q_{j},p_{j}$, not only the *n* first. It follows that the motion of the subsystem $\left(q^{\prime },p^{\prime }\right)$ is not governed by a Hamiltonian; this can be easily seen by finding the explicit solutions for simple systems. Let us illustrate this using Sharov’s argument [12]. Suppose Equation (4) represents the time-evolution of a bona fide Hamiltonian system. Denoting by $q_{1}^{0},...,q_{n}^{0};p_{1}^{0},...,p_{n}^{0}$ any set of initial conditions we have

$$\int |J\left(t\right)|dp_{1}^{0}\cdot \cdot \cdot dp_{n}^{0}dq_{1}^{0}\cdot \cdot \cdot dq_{n}^{0}=\int dp_{1}\cdot \cdot \cdot dp_{n}dq_{1}\cdot \cdot \cdot dq_{n}$$

where J(t) is the Jacobian of the transformation from the initial conditions to $\left(q^{\prime },p^{\prime }\right)$. If the system Equation (4) is Hamiltonian, then this transformation must be canonical, so we should have |J(t)|=1. However, we have, as Sharov [12] showed, dJ(t)/dt≠0, hence J(t)≠J(0)=1. In fact, the principle of the symplectic camel which we discuss below implies, without any calculation at all, that |J(t)|≥1. Thus:

*A subsystem of a Hamiltonian system is usually not a Hamiltonian system in its own right*.

What about the return time? A first educated guess is that since the subsystem interacts (perhaps very strongly) with the rest of the system this interaction will influence the return time which will become much longer than in the interaction-free case, perhaps even of the order $T\approx {\left(L/\varepsilon \right)}^{6N}$, at which the total system returns.

### 2.2. Non-Squeezing and Packing

Liouville’s theorem tells us that Hamiltonian motions are volume preserving: this is one of the best known results from elementary mechanics. However, in addition to being volume-preserving, Hamiltonian motions have unexpected “rigidity properties”, which distinguish them from ordinary volume-preserving diffeomorphisms. The most famous is Gromov’s *non-squeezing theorem.* Assume that we are dealing with a Hamiltonian system consisting of a large number *N* of particles with coordinates $q_{i}=\left(x_{i},y_{i},z_{i}\right)$ and momenta $p_{i}=\left(p_{x_{i}},p_{y_{i}},p_{z_{i}}\right)$. If these points are sufficiently close to each other, we may, with a good approximation, identify that set with a “cloud” of phase space fluid; by phase space we mean the space $R_{q,p}^{6N}$ with $q=(q_{1},q_{2},...,q_{N})$ and $p=(p_{1},p_{2},...,p_{N})$. Suppose that this cloud contains at time t=0 a ball with radius *R*:

(5) $$B_{R}:|q-q_{0}{|}^{2}+{\left|p-p_{0}\right|}^{2}\leq R^{2}.$$

The orthogonal projection of the cloud of points on any plane of coordinates $\left(x,p_{x}\right)$, $\left(x,p_{y}\right)$, $\left(x,p_{z}\right)$, etc. will thus have area at least $\pi R^{2}$. Let us now watch the motion of this phase-space cloud. As time evolves, it will distort and may take after a while a very different shape, while keeping constant volume in view of Liouville’s theorem. However—and this is the surprising result—the projections of that deformed cloud on any of the planes of *conjugate* coordinates $\left(x,p_{x}\right)$, $\left(y,p_{y}\right)$ or $\left(z,p_{z}\right)$ will never decrease below the value $\pi R^{2}$. This fact is of course strongly reminiscent of the uncertainty principle of quantum mechanics, of which it is in fact a classical version; we have discussed this analogy in detail in de Gosson [4,10]. Scheeres et al. [9] used our results to study orbit uncertainty in space craft navigation.

The phenomenon described above seems at first sight to conflict with the usual conception of Liouville’s theorem: according to folk wisdom, the ball $B_{R}$ can be stretched in all directions, and eventually get very thinly spread out over huge regions of phase space, so that the projections on any plane could a priori become arbitrary small after some time *t*; this stretching generically increases with time. In fact, one may very well envisage that the larger the number *N* of degrees of freedom, the more that spreading will have chances to occur since there are more and more directions in which the ball is likely to spread! This possibility has led to many quasi-philosophical speculations about the stability of Hamiltonian systems (in [4,5] we have discussed Penrose’s claim ([13], p. 183, l.–3) that phase space spreading suggests that “*classical mechanics cannot actually be true of our world*”). However, the phenomena we shortly described above show that such statements (which abound in the literature) come from a deep misunderstanding of the nature of Hamiltonian mechanics. The non-squeezing theorem prevents anarchic and chaotic spreading of the ball in phase space which would be possible if it were possible to stretch it inside arbitrarily thin tubes in directions orthogonal to the conjugate planes. This possibility is perfectly consistent with Katok’s lemma [14], which can be stated as follows: consider two bounded domains Ω and $\Omega^{\prime }$ in $R^{2n}$ which are both smooth volume preserving deformations of the ball $B_{R}$. Then, for every ε>0, there exists a Hamiltonian function *H* and a time *t* such that $\mathrm{Vol}(f_{t}^{H}\left(\Omega \right)\Delta \Omega^{\prime })<\varepsilon$. Here, $f_{t}^{H}\left(\Omega \right)\Delta \Omega^{\prime }$ denotes the set of all points that are in $f_{t}^{H}\left(\Omega \right)$ or $\Omega^{\prime }$, but not in both. Katok’s lemma thus shows that up to sets of arbitrarily small measure ε any kind of phase-space spreading is a priori possible for a volume-preserving flow, because $f_{t}^{H}\left(\Omega \right)$ can become arbitrarily close to $\Omega^{\prime }$.

The properties outlined above are best understood (and proved) in terms of a new generation of theorems from symplectic topology, the first of which goes back to the mid-1980s, and is known as *Gromov’s non-squeezing theorem* [15], which is often referred to as the *principle of the symplectic camel* (for various interpretations of this Biblical metaphor, see the comments to the online version of Reich’s review [16] of my paper [4]. This theorem—whose implications to physics have not yet been fully explored—has allowed us to give a symplectically invariant topological version of the principle of quantum indeterminacy [3,4,5], and to describe in a precise classical uncertainties arising in some systems [10].

### 2.3. One Step Further: Subsystems

Gromov’s non-squeezing theorem can be reformulated rigorously as follows: let *f* be a canonical transformation (often called a symplectic diffeomorphism, or symplectomorphism in the mathematical literature). This means that, if $\left(q^{\prime },p^{\prime }\right)=f\left(q,p\right)$, then the Jacobian matrix

(6) $$Df\left(q,p\right)=\frac{\partial (q^{\prime },p^{\prime })}{\partial (q,p)}$$

is symplectic, and thus, for every point (q,p) in $R^{6N}$ (Arnol’d [17]): Df(q,p)∈Sp(3N). Gromov’s theorem says that

(7) $$\mathrm{Area}\Pi_{j}\left(f\left(B_{R}\right)\right)\geq \pi R^{2}$$

where $\Pi_{j}$ is the orthogonal projection on the plane of conjugate variables $\left(q_{j},p_{j}\right)$; here the index j is any of the integers 1,...,N. We now address the following more general question: is there a generalization of this result to higher dimensional subspaces of $R^{6N}$? More specifically, what we have in mind is the volume of the projection $\Pi^{\prime }$ of $f(B_{R})$ on a subspace $R^{6n}$ in the conjugate coordinates $\left(q^{\prime },p^{\prime }\right)=\left(q_{1},...,q_{3n},p_{1},...,p_{3n}\right)$; 1<n<N. Such a subspace of $R^{6N}$ inherits in a natural way a symplectic structure, and we ask: is the “obvious” generalization

(8) $${\mathrm{Vol}}_{6n}\Pi^{\prime }\left(f\left(B_{R}\right)\right)\geq \frac{\pi^{3n}}{(3n)!}R^{6n}$$

of Equation (7) true? Let us call a canonical transformation satisfying the property in Equation (8) a “hereditary” canonical transformation. It has been very recently proved by Abbondandolo and Matveyev [18] that:

*All linear (or affine) canonical transformations are hereditary.*

An immediate consequence of this fact is that if $\left(f_{t}^{H}\right)$ is the flow determined by the Hamilton equations for a Hamiltonian function of the type

$$H\left(q,p\right)=\sum_{j=1}^{3N}a_{j}\left(t\right)p_{j}^{2}+b_{j}\left(t\right)q_{j}^{2}+c_{j}\left(t\right)p_{j}+d_{j}\left(t\right)q_{j}$$

where $a_{j},b_{j},c_{j},d_{j}$ are continuous real functions of the time *t*, then each $f_{t}^{H}$ is hereditary. This applies, in particular to the independent oscillator model of a heat bath where

$$H\left(q,p,x,p_{x}\right)=\frac{1}{2m}p_{x}^{2}+Kx^{2}+\sum_{j=1}^{3N}\frac{1}{2m_{j}}\left(p_{j}^{2}+m\omega_{j}^{2}{\left(x-q_{j}\right)}^{2}\right)$$

is the Hamilton function of a linear oscillator in the $\left(x,p_{x}\right)$ variables coupled with *N* isotropic oscillators with frequencies $\omega_{1},...,\omega_{N}$.

Let us discuss the non-linear case. In [18], the authors constructed a counterexample showing that there exist nonlinear canonical transformations which are not hereditary. However, the transformation they constructed deforms the ball $B_{R}$ tremendously and seems to be very unphysical. Now, in the same paper, Abbondandolo and Matveyev discussed the validity of Equation (8) for more general canonical transformations when the radius *R* is small; they conjecture that this property is generically true of all Hamiltonian systems. At the time of writing, there is however no convincing proof of this conjecture. We are thus in the unusual (and unpleasant) situation where we would like to use a theorem valid for a class of Hamiltonians which has not, at the time of writing, been fully characterized! (However, see the comments in Schlenk’s review paper [19]). If true, there would be large subclasses of Hamiltonian (sub)systems exhibiting superrecurrence.

### 2.4. A Simple Case of Superrecurrence

As was pointed out by Polterovich (see Schlenk [19]), the original motivation for Gromov to study “packing numbers” in symplectic topology was his search for recurrence properties which are stronger than those of volume preserving mappings. Consider first the following simple planar situation: we have a disk $D_{R}\left(0\right)$ in the plane $R^{2}$, that is, the set of points $\left(x,p_{x}\right)$ such that $x^{2}+p_{x}^{2}\leq R^{2}$. We have $\mathrm{Area}\left(D_{R}\left(0\right)\right)=\pi R^{2}$. We now ask the question: For which radius *r* can we embed two smaller *disjoint* disks $D_{r}\left(a\right)$ and $D_{r}\left(b\right)$ inside $D_{R}\left(0\right)$ using a canonical transformation? The answer is easy: in the plane, canonical transformations are just the area preserving diffeomorphisms, so it suffices that

$$\mathrm{Area}\left(D_{r}\left(a\right)\right)+\mathrm{Area}\left(D_{r}\left(b\right)\right)\leq \mathrm{Area}\left(D_{R}\left(0\right)\right)$$

that is, $2r^{2}\leq R^{2}$. We can thus embed at best two disjoint disks $D_{R/\sqrt{2}}\left(a\right)$ and $D_{R/\sqrt{2}}\left(b\right)$ inside a disk with radius *R*, and that disk is then completely filled by the images of deformed smaller disks. Choose now a general phase space $R^{2n}$ (with for instance n=3N) and consider the same problem for a ball $B_{R}\left(0\right)$ with radius *R*. We want to pack two smaller disjoint balls $B_{r}\left(a\right)$ and $B_{r}\left(b\right)$ inside $B_{R}\left(0\right)$ using general canonical transformations. Calculating the volumes, we have

$${\mathrm{Vol}}_{2n}\left(B_{R}\right)=\frac{{\left(\pi R^{2}\right)}^{n}}{n!}\;\;,\;\;{\mathrm{Vol}}_{2n}\left(B_{r}\right)=\frac{{\left(\pi r^{2}\right)}^{n}}{n!}$$

and hence

(9) $${\mathrm{Vol}}_{2n}\left(B_{R}\right)/{\mathrm{Vol}}_{2n}\left(B_{r}\right)={\left(R/r\right)}^{2n}.$$

This indicates that, at first sight, we could pack $2^{n}$ balls with radius $r=R/\sqrt{2}$ inside $B_{R}\left(0\right)$ and this number becomes very large when the number *n* of degrees of freedom increases. The reality is, however, very different. The argument above, while true for arbitrary volume preserving diffeomorphisms, does not take into account the fact that we are dealing here with canonical transformations, and that the latter are much more “rigid” than ordinary volume preserving diffeomorphisms. In fact, when we are dealing with canonical transformations (and hence, in particular, Hamiltonian flows), the following result holds: 

Gromov’s Two Balls Theorem [15]: *If two disjoint phase space balls with radiusrare mapped inside a ball* $B_{R}$ *by a canonical transformation, then we must have* $r\leq R/\sqrt{2}$*.*

In other words, there is no quantitative difference between the packing number in two-dimensional phase plane and a general phase space $R^{2n}$ with n>1. This implies that any obstruction to symplectically embedding a ball into a larger ball is much stronger than the volume constraint given by Equation (9). This is a very strong result, and allows proving a simple superrecurrence theorem: assume that we have a “Universe” U that is the image of a very large ball $B_{R}$ by some canonical transformation (it may be a symplectic ellipsoid, or more generally any compact symplectic manifold with boundary). Take a subset B of U which is the image of a ball with radius $\left(R+\varepsilon \right)/\sqrt{2}$ where ε is a small number, say ε=(1/n)R. Then,

(10) $${\mathrm{Vol}}_{2n}\left(U\right)/{\mathrm{Vol}}_{2n}\left(B\right)=N\approx e^{-2}2^{n}.$$

Now, let *H* be a Hamiltonian function whose flow $\left(f_{t}^{H}\right)$ preserves the universe U, that is $f_{t}^{H}\left(U\right)$ (it is sufficient that the Hamiltonian vector field $X_{H}$ is tangent to the boundary ∂U). The flow $f_{t}^{H}$ displaces the “subuniverse” B which becomes $f_{t}^{H}\left(B\right)$ after time *t*. If we only use the fact that $f_{t}^{H}$ volume preserving; then Equation (10) would imply that the recurrence time could be very large: choosing t=1 as a unit of time and setting $f=f_{t}^{H}$ the sets f(B), $f^{2}\left(B\right)$,...., $f^{N-1}\left(B\right)$ cannot be all disjoint, and hence the return time can a priori be as large as N-1. However, by Gromov’s Two Balls Theorem, we will have f(B)∩B≠∅ so the first return time is t=1.

**Remark** **1.** *Gromov’s Two Balls Theorem’ whose classical consequences we discussed above is related to the notion of dislocation of quantum states as discussed by Polterovich [20] and Charles and Polterovich [21].*

## 3. Discussion

I have discussed in this contribution to EMQM17 some consequences of symplectic topology on Poincaré recurrence from a perfectly classical point of view. However, as I have explained elsewhere with Basil Hiley [6], the properties of symplectic topology described here are reminiscent of certain aspects of quantum mechanics (for instance, the uncertainty principle). I view them as *imprints of the quantum world* on classical mechanics in its Hamiltonian formulation. Of course, this point of view might be felt as controversial by some physicists, so let me explain what I have in mind (thus, partially answering some interesting remarks and objections made by a Referee). In either of its formulations, quantum mechanics is built on classical mechanics. In the Heisenberg picture, one wants to give an operator-theoretical meaning to Hamilton’s equations of motion

$$\frac{dq}{dt}=\frac{\partial H}{\partial p}\left(q,p,t\right)\;\;,\;\;\frac{dp}{dt}=-\frac{\partial H}{\partial q}\left(q,p,t\right)$$

and this is done by replacing the classical position and momentum variables *q* and *p* with operators $\hat{q}$ and $\hat{p}$ satisfying the Born condition $[\hat{q},\hat{p}]=i\hbar$; after some work, one is led to the quantum Hamilton equations

(11) $$\frac{d\hat{q}}{dt}=\frac{\partial H}{\partial p}\left(\hat{q},\hat{p},t\right)\;\;,\;\;\frac{d\hat{p}}{dt}=-\frac{\partial H}{\partial \hat{q}}\left(\hat{q},\hat{p},t\right).$$

In the Schrödinger picture, which is based on de Broglie’s wave mechanics, the time-evolution of the wavefunction ψ is governed by Schrödinger’s equation

(12) $$i\hbar \frac{\partial \psi }{\partial t}=\hat{H}\psi$$

where $\hat{H}$ is an operator associated with the classical Hamiltonian function *H*. Now, it is often claimed that both pictures lead to the same physical predictions. However, this is only true if one uses the same quantization procedure for both the Heisenberg and Schrödinger theories. In fact, the quantum Hamilton equation (Equation (11)) only make sense if one quantizes products $q^{m}p^{n}$ using the prescriptions given in 1923 by Born and Jordan, together with Heisenberg, in their famous “Dreimännerarbeit”; it follows that the operator $\hat{H}$ in Schrödinger’s Equation (12) must be derived from the Hamiltonian function *H* also using the Born–Jordan prescription (for otherwise both pictures would no longer be equivalent, as I have discussed in detail in [22,23]). This short digression is intended to explain that, no matter how one “defines” quantum mechanics, the classical (Hamiltonian) theory is always present as a watermark. In fact, in my opinion, this is a quite logical consequence of the fact that we, humans, are macroscopic objects and as such the only direct experience we have from our World is of a macroscopic nature, and there is no “pedagogical” way to reverse this approach, that is to make us in first place become aware of the quantum nature of our environment, and then to deduce the classical properties as an approximation thereof. Thus, all this brings me to the following observation: quantum physics is a mathematical construct. However, mathematics is an exact Science; there is no place for polemic, interpretations, or controversy. Mathematics is not as “emotional” as physics is: a mathematical statement is either *true*, or it is *false*. It turns out that I have shown in [24] that symplectic geometry is the common mathematical background of classical and quantum mechanics (also see our paper with Hiley [6]), and that both theories are mathematically *equivalent*. The proof mainly relies on the fact that a Hamiltonian isotopy automatically generates a quantum isotopy (it is a consequence of the theory of the metaplectic group) and vice versa to every quantum isotopy we can associate a Hamiltonian isotopy. This property shows that there is a *canonical* isomorphism between quantum and classical theory. A *caveat* here: the Reader is invited to observe that this is by no way a provocative or paradoxical statement: it is just a mathematical theorem, which may be perceived as counterintuitive by many physicists. Now, a mathematical theory has no a priori physical meaning unless one creates an interpretational apparatus allowing to draw real-life consequences from the mathematical objects: an equation is not a physical theory! This explains the statement I made above, namely that “...properties of symplectic topology ... can be viewed as *imprints of the quantum world* on classical mechanics...”. If both the quantum and classical theory are mathematically equivalent, the sentence could indeed be reversed by saying that it is classical mechanics which leaves imprints on the quantum world. However, from the physical point of view, we are in a “Cheshire cat” scenario: since experience shows that quantum mechanics yields a better description of Nature than classical mechanics, quantum theory contains classical mechanics as an approximation, and leaves in this approximation some features reminiscent of the true theory, exactly as when the legendary cat disappears but leaves his grin.
